# A Large Language Model–Driven System for Advance Care Planning Training Among Health Care Providers in the Chinese Context: Development and Technical Evaluation

**DOI:** 10.2196/87288

**Published:** 2026-07-28

**Authors:** Minghui Tan, Siyuan Tang, Shichao Kan, Bei Wu, Zhao Ni, Haojie Zhang, Jinfeng Ding

**Affiliations:** 1 Xiangya School of Nursing, Central South University, 172 Tongzipo Road, Changhsa, Hunan, 410013, China, 86 18711000745; 2 School of Nursing, Ningxia Medical University, Yinchuan, Ningxia, China; 3 School of Computer Science and Engineering, Central South University, Changsha, Hunan, China; 4 New York University Shanghai, Shanghai, Shanghai, China; 5 School of Nursing, Yale University, New Haven, CT, United States

**Keywords:** advance care planning, health care providers, large language model, multi-agent, chatbot, synthetic data, artificial intelligence, AI

## Abstract

**Background:**

With the expanding need for advance care planning (ACP), innovative educational strategies for training health care providers are increasingly required. Large language model (LLM)–based ACP chatbots offer a novel and potentially effective solution to enhance health care providers’ competence in navigating complex ACP conversations.

**Objective:**

This study aimed to develop a Chinese-context ACP corpus to support an LLM-based chatbot and evaluate the feasibility and performance of a multi-agent system for simulating complex ACP discussions as a training tool for health care providers.

**Methods:**

This study involved dataset construction and model adaptation and evaluation. We constructed 3 structured datasets using synthetic dialogue data generated through prompts derived from ACP-related scientific literature and policy documents. Both open-source (Zhongjing) and closed-source LLMs (GPT-4o-mini) were chosen as baseline models. The Zhongjing model was adapted through fine-tuning, whereas GPT-4o-mini was adapted using both fine-tuning and prompt engineering. Model performance was assessed through automatic and human evaluations following the QUEST (Quality of information, Understanding and reasoning, Expression style and persona, Safety and harm, and Trust and confidence) framework. Statistical comparisons between baseline and adapted models were performed using repeated-measures ANOVA.

**Results:**

Three separate datasets for the assistant, vignette, and evaluator agents were created, which collectively formed a multi-agent artificial intelligence system for Chinese ACP training. The assistant dataset included 4364 dialogues, the vignette dataset comprised 671 clinical scenarios, and the evaluator dataset contained 671 records. Both automatic and human evaluations confirmed that the adapted models significantly outperformed baseline models on most aspects of Chinese ACP conversations and summarization (η^2^_p_=0.12‐0.99; *P* values ranged from .03 to <.001).

**Conclusions:**

This study demonstrates the adequate technical feasibility of the multi-agent LLM-based system for ACP training among health care providers in the Chinese context. Despite its potential as a supportive educational tool, further validation in real-world training contexts is required to establish its effectiveness in enhancing health care providers’ ACP competencies.

## Introduction

### Background

Advance care planning (ACP) is a critical process that empowers individuals to articulate their values, life goals, and preferences regarding future health care decisions [1]. Effective ACP often involves complex and sensitive conversations among health care providers, patients, and their families [2]. Numerous systematic reviews have consistently shown that ACP offers various advantages for health care systems, patients, families, and health care providers, including improved patient satisfaction, reduced decision-making burden on families, and better alignment between patient wishes and the care provided [3-5].

Despite these documented benefits, ACP discussions are often fraught with challenges, especially for health care providers, who serve as initiators and facilitators of ACP. Health care providers must navigate complex, emotionally charged, and sometimes culturally sensitive conversations with patients and their families [6]. These discussions often involve confronting difficult topics such as prognosis, end-of-life decisions, and personal values, which can evoke strong emotional responses and lead to communication breakdowns [7]. Furthermore, variations in cultural norms, family dynamics, and individual beliefs can complicate the process, making it difficult for health care providers to foster an open, respectful, and effective dialogue [8]. Although ACP training opportunities are available for health care providers [9,10], offline training requires substantial resources and time commitment from both instructors and trainees, making it challenging amid busy clinical schedules [11]. Moreover, participants’ feedback has highlighted a desire for more practical training formats incorporating experiential learning approaches [12]. In contrast, online trainings often lack interactivity, limiting engagement, intellectual stimulation, and opportunities for confrontational learning experiences [11]. In light of these challenges, there is a growing need to explore innovative training methods that can better equip health care providers to conduct ACP discussions with skill and confidence.

Recent advances in artificial intelligence (AI) have created new opportunities to enhance ACP training. One of the most promising developments is the application of large language models (LLMs) in health care education [13-15]. LLMs, such as OpenAI’s generative pretrained transformer–based systems, can generate coherent, contextually appropriate responses to complex prompts. However, the current research landscape on LLMs in the ACP domain has primarily focused on their role in assisting with clinical record extraction—identifying, extracting, and analyzing relevant information from medical documents and patient records [16,17]. While this is a valuable application, it does not fully leverage LLMs’ capability to generate humanlike language. LLMs can give health care providers an opportunity to rehearse difficult discussions, receive instant feedback, and refine their approaches based on simulated patient responses. This iterative learning process will be a vital complement to previous ACP training methods, which may not offer the same level of repeatable and personalized engagement. To date, there is a notable gap in the literature regarding the development of LLM-based tools for simulating nuanced ACP conversations.

### Objectives

This study aimed to address this gap by introducing an innovative LLM-based ACP chatbot designed to improve health care providers’ competence and uptake for complex ACP discussions. This study had 2 objectives. First, it described the development of an ACP corpus to adapt LLMs, focusing on creating high-quality, contextually appropriate datasets of interactive ACP. Second, it evaluated the chatbot’s performance in a Chinese context, comparing it to existing baseline models and various adaptation approaches.

## Methods

### Overview

**Figure 1.**
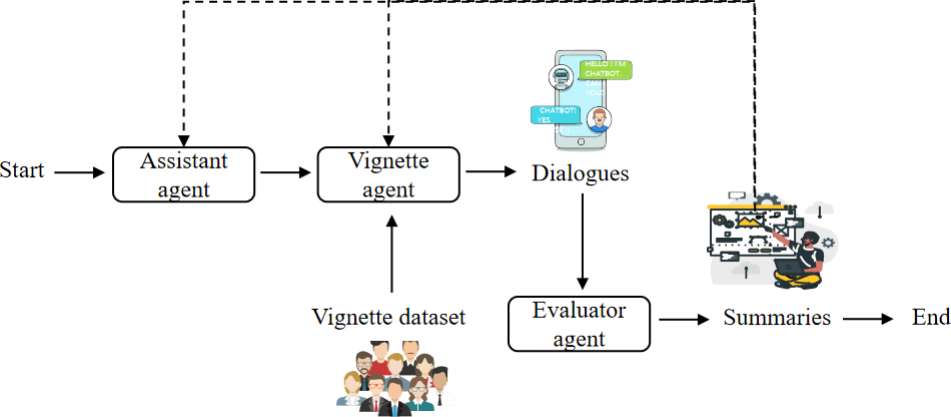
Multi-agent artificial intelligence system for advance care planning (ACP): the assistant agent facilitates ACP training, the vignette agent simulates patient or family scenarios, and the evaluator agent provides feedback on ACP discussions. Evaluation results are fed back to the assistant and vignette agents to support iterative refinement of model behavior.

The LLM-based ACP chatbot was implemented as a multi-agent system composed of 3 specialized agents [18] (Figure 1), each aligned with a distinct component of the ACP training process. The *assistant agent* supported users’ conceptual learning by introducing ACP principles and responding to questions. The *vignette agent* simulated patients or family members in interactive role-play scenarios to allow users to practice ACP dialogue in a realistic conversational setting. The *evaluator agent* provided structured feedback by analyzing user-vignette interactions and identifying strengths and areas for improvement. This modular design enabled clear functional separation and iterative learning support. The development process involved 3 phases: constructing ACP-specific datasets, adapting LLMs using these data, and evaluating performance. This study was reported in accordance with the DEAL (Development, Evaluation, and Assessment of Large Language Models) checklist [19].

### Data Collection and Dataset Construction

**Figure 2.**
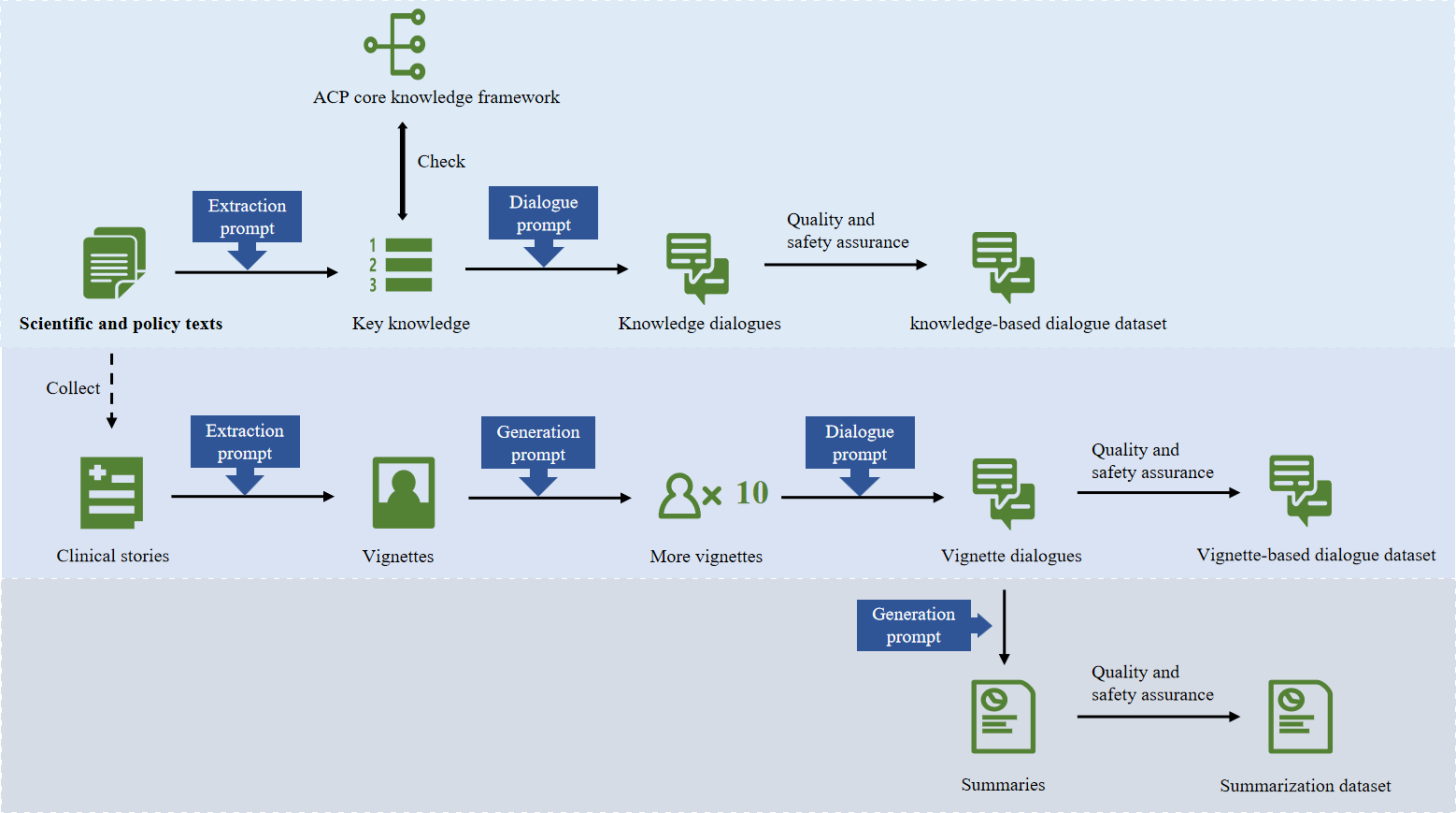
Dataset construction pipeline: English- and Chinese-language advance care planning (ACP) texts were systematically collected using GPT-4o with task-specific prompts and converted into knowledge dialogues, vignette dialogues, and summaries followed by quality and safety assurance.

We systematically collected scientific and policy documents on ACP from academic databases and official websites from database inception to March 2024 to build the ACP datasets. As ACP discussions involve sensitive end-of-life decision-making and vulnerable patient populations, collecting authentic dialogues would require complex ethics approvals, multisite coordination, and extensive deidentification procedures [20]. High-quality, structured ACP dialogue datasets are rarely available publicly, particularly in the Chinese health care context. Furthermore, there is emerging evidence suggesting that synthetic data can approximate real-world data in terms of performance and structural characteristics [21]. Therefore, we primarily used synthetic data generated via prompt-based methods. A prompt [22] served as an input instruction that guided the model—specifically, GPT-4o (OpenAI) in this study—on how to respond, shaping its output in accordance with the designated task. Different datasets were prepared for each agent: (1) a knowledge-based dialogue dataset for the assistant agent, (2) a vignette-based dialogue dataset for the vignette agent, and (3) a summarization dataset for the evaluator agent (Figure 2).

The development of a knowledge-based dialogue dataset involved several steps. First, ACP-related literature—including clinical guidelines, evidence summaries, systematic reviews, and Chinese legal and policy documents—was systematically searched and collected from relevant databases and official government websites. Inclusion criteria required that sources be directly relevant to ACP or end-of-life care practices and available in English or Chinese. Exclusion criteria eliminated sources that were abstract-only records, articles without evidence support, or duplicates across databases. Search strategies combined terms related to ACP, advance directives, end-of-life care, and palliative care. Second, GPT-4o with a predefined extraction prompt (Multimedia Appendix 1; original text in Chinese translated into English) was used to distill key knowledge from the collected ACP-related literature. The extracted knowledge was checked against the ACP core knowledge framework [23] to ensure completeness, with any identified omissions supplemented. Finally, a dialogue prompt was applied to produce knowledge-grounded ACP dialogues.

The development of vignette-based dialogue followed a structured process. Initially, clinical cases were gathered from the ACP-related literature mentioned above. GPT-4o, guided by an extraction prompt, was then used to extract essential details for vignette creation. Each vignette included essential demographic variables, ACP readiness stages, behavioral determinants, and intervention strategies. Subsequently, a generation prompt adapted from the vignette generation methodology [24] was applied to systematically generate approximately 10 distinct ACP scenarios for each base case. The generation protocol incorporated theoretically defined variation across demographic attributes, clinical factors, ACP readiness stages, behavioral determinants, and intervention strategies while explicitly prohibiting duplication of key behavioral and intervention elements within the same base case. Next, simulated dialogues between health care providers and patients or family members were created using a dialogue prompt in GPT-4o.

The summarization dataset was derived from the vignette-based dialogue dataset. Guided by a generation prompt, GPT-4o was used to analyze the strengths and limitations of the dialogues in a “summary-detail-summary” format. In this format, the evaluator first provided an overall summary of performance followed by a detailed enumeration of key strengths and areas for improvement and concluded with a closing summary that highlighted actionable guidance for future development.

Quality and safety assurance involved a multilayered evaluation process. All generated datasets underwent structured data processing conducted by trained graduate researchers, including anonymization, grammatical and logical error correction, medical terminology standardization, and removal of irrelevant symbols. To further enhance quality and reduce circular evaluation bias, an independent LLM (Qwen2.5, developed by Alibaba Cloud) [25] was used as an external evaluator. Knowledge-based dialogue and summarization datasets were rated across empathy, safety, readability, helpfulness, and factual accuracy using a 5-point scale; samples scoring below 3 on any dimension were manually revised and re-evaluated. The vignette-based dialogue dataset was assessed using the 10-item Decision Support Analysis Tool [26], and samples scoring below 6 were similarly revised.

The dataset was randomly divided into training, validation, and test sets using an 80%-10%-10% split at the conversation (vignette) level. All dialogue turns belonging to a given conversation were assigned exclusively to a single partition to avoid potential data leakage. The training set was used for fine-tuning, the validation set was used for hyperparameter optimization and overfitting monitoring, and the held-out test set was used for unbiased performance evaluation. The assistant agent was trained to generate responses at the turn level within conversations, whereas the vignette and evaluator agents operated at the vignette level.

### Model Selection

The selection of an appropriate pretrained model for fine-tuning is a critical step to ensure the robustness, adaptability, and efficacy of the resulting system. In this study, we used both an open-source LLM, Zhongjing, and a closed-source LLM, GPT-4o-mini.

Zhongjing [27] was selected for its transparency, adaptability, and extensive training on medical datasets. As an open-source model, its architecture, training code, and weights are publicly accessible, which allows researchers to adapt and optimize the model for different contexts. Compared with other open-source models, Zhongjing demonstrates particularly strong competence in medical knowledge and conversational ability due to its extensive training on Chinese medicine–related datasets [27]. The Zhongjing model was originally introduced in 2023 [28], and we used this version as the base model.

GPT-4o-mini [29], a compact variant of the GPT-4o architecture, was chosen for its state-of-the-art natural language understanding and generation capabilities. GPT-4o-mini represents a closed-source model, meaning that its internal architecture, pretraining data, and model weights are proprietary and not publicly disclosed. GPT-4o-mini excels in generating coherent, contextually appropriate responses and handling nuanced, emotionally sensitive dialogues, which are critical for ACP conversations. Its proprietary nature ensures optimized performance, robust pretraining, and continued updates, contributing to its reliability in handling complex linguistic tasks. The version used in this study corresponded to the version released on July 18, 2024 (gpt-4o-mini-2024-07-18).

### Model Adaptation

The adaptation of pretrained models to the ACP domain used 2 conceptually distinct strategies: fine-tuning and prompt engineering. Detailed adaptation configurations are provided in Multimedia Appendix 2.

Fine-tuning a language model involves adjusting a pretrained model by training it on smaller, task-specific data. For Zhongjing, we used low-rank adaptation (LoRA), which was among the most commonly used fine-tuning approaches for open-source LLMs. LoRA [30] was an efficient fine-tuning technique that modified the model by introducing low-rank matrices into specific parts of the neural network. LoRA was advantageous as it reduced the computational burden compared to traditional full fine-tuning, focusing only on a small set of parameters that required adjustment.

In contrast, GPT-4o-mini underwent supervised fine-tuning and prompt engineering. Supervised fine-tuning is the default fine-tuning method in the ChatGPT official website. Supervised fine-tuning [31] is a process where a pretrained model is further trained on a labeled dataset with specific task-oriented examples, enabling it to learn patterns and improve performance for particular tasks by adjusting its weights through gradient descent based on the labeled input-output pairs.

Prompt engineering [32] does not modify the model’s weights but, instead, relies on carefully designed instructions to guide a very large pretrained model to produce high-quality, contextually appropriate outputs. The key to this process was to build specialized agents based on different prompts to interact with users (Multimedia Appendix 1) through a stepwise reasoning process inspired by the chain-of-thought (CoT) methodology. For example, the vignette agent prompt instructs the model to reveal patient context progressively and interactively across multiple conversational steps, which mirrors the CoT principle of decomposing a task into intermediate reasoning steps before producing a response. While not a formal CoT prompt, this stepwise guidance encourages the model to reason and respond in a structured, contextually appropriate manner. This approach was applied only to GPT-4o-mini as Zhongjing currently does not support flexible prompt engineering in a systematic manner. Moreover, while all LLMs require input prompts to generate responses, in this study, the base and fine-tuning models were indeed provided with systems prompts to interact with users, and the fine-tuning models did not use CoT reasoning during inference.

### Evaluation Metrics

The performance of the models was assessed through both human and automatic evaluations supplemented by ablation studies. Ablation studies use a leave-out testing strategy where the impact of removing or modifying specific components is measured. The models analyzed were (1) GPT-4o-mini with fine-tuning and prompt engineering, (2) GPT-4o-mini with fine-tuning, (3) GPT-4o-mini with prompt engineering, (4) base GPT-4o-mini model, (5) Zhongjing with fine-tuning, and (6) base Zhongjing model.

Human evaluation was carried out by a panel of 11 health care providers with expertise in ACP, and their characteristics are detailed in Table 1. Prior methodological literature suggests that expert panels of approximately 10 to 15 members are generally adequate [33]. A review of studies on the human evaluation of LLMs in health care indicated that most studies involve between 1 and 20 evaluators [34]. This suggested that the panel size used in this study fell within the commonly reported range. For the human evaluation phase, we sampled fixed numbers of complete cases from each test subset to evaluate each agent. Specifically, we sampled 5 knowledge-based dialogues for the assistant agent, 10 vignette-based dialogue cases for the vignette agent, and 3 dialogue summary cases for the evaluator agent. Because the 3 evaluation tasks differed substantially in dialogue structure, interaction complexity, and annotation burden, the final sample sizes differed across subsets. All experts independently completed evaluations using standardized scoring instructions and were blinded to model type. Interrater reliability was assessed using the intraclass correlation coefficient based on a 2-way mixed-effects model with absolute agreement. The intraclass correlation coefficient values for each evaluation dimension ranged from 0.84 to 0.93, indicating good to excellent agreement among the experts.

**Table 1.** Demographic characteristics of the human experts.

Characteristic	Values
Sex, n (%)	
Female	10 (90.9)
Male	1 (9.1)
Age (y), mean (SD)	45.80 (11.6)
Educational level, n (%)	
Bachelor’s degree	3 (27.2)
Master’s degree	4 (36.4)
PhD	4 (36.4)
Profession type, n (%)	
Nurse	5 (45.4)
Academic	4 (36.4)
Physician	1 (9.1)
Lawyer	1 (9.1)
Research or practice field, n (%)	
Palliative care	9 (81.8)
Health law	1 (9.1)
Critical care	1 (9.1)
Research or practice experience (y), mean (SD)	18.09 (14.6)
Familiarity with ACP^a^, n (%)	
Familiar	5 (45.4)
Very familiar	6 (54.6)

a ACP: advance care planning.

The GPT-4o model was used for automatic evaluation on the test datasets because it represented one of the most advanced available LLMs, with state-of-the-art performance in instruction following, clinical language understanding, and rubric-based evaluation. The quality of the responses generated by each ablated model on the test set was evaluated according to the QUEST framework [34], which incorporates five distinct evaluation dimensions: (1) quality of information (accuracy, relevance, and completeness of content), (2) understanding and reasoning (the model’s ability to correctly interpret context and provide logical responses), (3) expression style and persona (clarity, coherence, and alignment with the intended professional persona), (4) safety and harm (sensitivity to potential risks, bias, or harmful content), and (5) trust and confidence (the degree to which users can rely on and feel confident in the model’s outputs). Each of these dimensions was rated on a 5-point scale, allowing for a detailed analysis of the models’ performance across multiple facets in generating responses.

Statistical analyses were performed using SPSS (version 26; IBM Corp). The evaluation scores, collected using 5-point Likert scales, were treated as approximately continuous variables based on their near-normal distribution, as indicated by normality tests and consistent with common statistical practice [35,36]. Because all models were evaluated on the same set of test datasets, a repeated-measure ANOVA was conducted to compare model performance while accounting for within-dataset dependence. The assumption of sphericity was assessed using the Mauchly test, and where violated, the Greenhouse-Geisser correction was applied. Pairwise comparisons were performed using the Bonferroni correction. Effect sizes were calculated and reported as η^2^_p_. *P* values of less than .05 were considered to be statistically significant in all tests.

### Ethical Considerations

The research was approved by the Ethical Review Board of Xiangya School of Nursing, Central South University (approval E202442; May 6, 2024). All participants were asked to provide written informed consent before interacting with the AI system and were briefed on the study’s objectives and procedures. Participants received a small monetary incentive valued at approximately ¥200 (RMB ¥1=US $0.14 as of April 30, 2025) as compensation for their time and participation. To protect privacy and confidentiality, all collected data were anonymized and deidentified prior to analysis. No identifying information is reported in this manuscript or in any supplementary materials.

## Results

### Datasets

#### Knowledge-Based Dialogue Dataset

A total of 2170 ACP-related documents were systematically retrieved, and 415 (19.1%) were finally included, encompassing 3 (0.7%) computerized decision support systems, 63 (15.2%) guidelines, 18 (4.3%) synopses, 275 (66.3%) systematic reviews, and 56 (13.5%) policy articles. Through the iterative process of knowledge extraction and supplementation, 4217 knowledge points were extracted. The evaluation results showed that the coverage rate of the ACP core knowledge framework reached 100% (Table S1 in Multimedia Appendix 3). Under the guidance of dialogue prompts, a total of 4364 dialogues were generated (Table 2).

**Table 2.** Characteristics of the advance care planning datasets.

Dataset	Samples, n	Rounds per sample, mean (SD)	Word count per dialogue round, mean (SD)	Total dialogue rounds, n	Total character count, n
Knowledge-based dialogue dataset	4364	3.29 (0.73)	32.12 (17.24)	14,367	461,460
Vignette-based dialogue dataset	671	29.12 (9.59)	58.82 (41.98)	19,539	1,149,224
Summarization dataset	671	2.00 (0)	1677.00 (744.28)	1342	2,250,530

#### Vignette-Based Dialogue Dataset

We collected a total of 61 clinical cases from the retrieved articles (Table S2 in Multimedia Appendix 3). The demographics of the patients depicted in the vignettes showed that most were aged 60 to 79 years, with a roughly equal ratio of men to women. Most vignettes did not mention race and religious beliefs, and the diagnoses were mostly cancer. In terms of the transtheoretical model, most cases were in the precontemplation stage for “health care proxy” and “decision-making flexibility,” whereas most were in the action stage for “what is most important in life.” The behavioral drivers of personal characteristics, cognitive biases, and attitude self-efficacy and the behavior change techniques of goals and planning and shaping knowledge appeared frequently. On the basis of the generation prompts, more diverse vignettes were then generated, and the key characteristics are also described in Table S2 in Multimedia Appendix 3. From these vignettes, 671 simulated dialogues were generated using dialogue prompts, constructing multiple rounds of health care provider–patient or family member interactions (Table 2 and Table S3 in Multimedia Appendix 3).

#### Summarization Dataset

A total of 671 summarizations were generated based on the vignette-based dialogue dataset, with an average of 3.99 (SD 0.26) strengths and 3.10 (SD 0.15) limitations mentioned per summarization (Table S4 in Multimedia Appendix 3). Basic communication skills, ACP-related knowledge, and the application of behavior change techniques were the 3 most common advantages. Nevertheless, both ACP-related knowledge and behavior change techniques were found to be insufficient in some cases.

### Evaluation

**Figure 3.**
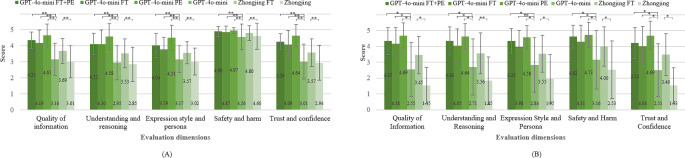
Automatic evaluation (A) and human evaluation (B) of the assistant agent. Bar heights represent mean scores, and error bars indicate SDs. **P<.01; *P<.05; FT: fine-tuning; PE: prompt engineering.

The evaluation results for the assistant agent models demonstrated that across most dimensions, the adapted models consistently outperformed the baseline models both for open-source and closed-source models and under both human and automatic evaluation (Figure 3; η^2^_p_=0.12‐0.90; Table S5 in Multimedia Appendix 3). However, the evaluation outcomes of different adaptation processes varied depending on the evaluators. In human evaluation, GPT-4o-mini with prompt engineering scored substantially higher than GPT-4o-mini with fine-tuning in most dimensions except for quality of information and trust and confidence. Automatic evaluations revealed that GPT-4o-mini with prompt engineering outperformed both GPT-4o-mini with fine-tuning and prompt engineering and GPT-4o-mini with fine-tuning across the five evaluation dimensions: quality of information (4.65, SD 0.71 vs 4.35, SD 0.51 vs 4.19, SD 0.80), understanding and reasoning (4.58, SD 0.81 vs 4.12, SD 0.66 vs 4.10, SD 0.88), expression style and persona (4.51, SD 0.77 vs 4.04, SD 0.75 vs 3.79, SD 0.75), safety and harm (4.97, SD 0.18 vs 4.90, SD 0.30 vs 4.87, SD 0.37), and trust and confidence (4.64, SD 0.71 vs 4.26, SD 0.51 vs 4.09, SD 0.86).

**Figure 4.**
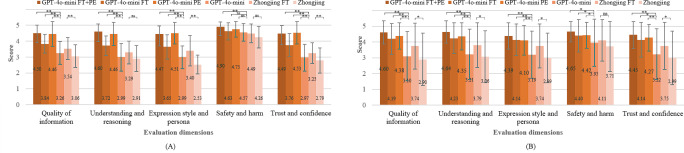
Automatic evaluation (A) and human evaluation (B) of the vignette agent. Bar heights represent mean scores, and error bars indicate SDs. **P<.01; *P<.05; FT: fine-tuning; ns: not significant; PE: prompt engineering.

Evaluation results for the vignette agent models revealed that the adapted models outperformed the baseline model across most dimensions. This superiority was evident both for open-source and closed-source models, as well as for evaluations performed by humans and automatic systems (Figure 4; η^2^_p_=0.18‐0.77; Table S6 in Multimedia Appendix 3). During the model adaptation process of GPT-4o-mini, both automatic and human evaluations demonstrated that the fine-tuning with prompt engineering strategy yielded the highest scores in most dimensions. Human evaluations indicated that GPT-4o-mini adapted with fine-tuning and prompt engineering substantially outperformed its fine-tuning–adapted counterpart across five dimensions: quality of information (4.60, SD 0.73 vs 4.19, SD 0.98), understanding and reasoning (4.64, SD 0.71 vs 4.23, SD 0.97), expression style and persona (4.39, SD 0.84 vs 4.14, SD 1.01), safety and harm (4.65, SD 0.64 vs 4.40, SD 1.02), and trust and confidence (4.45, SD 0.85 vs 4.14, SD 1.10). The automatic evaluation, on the other hand, showed that both the fine-tuning with prompt engineering and prompt engineering–only models scored substantially higher than the fine-tuning model in all dimensions except safety and harm.

**Figure 5.**
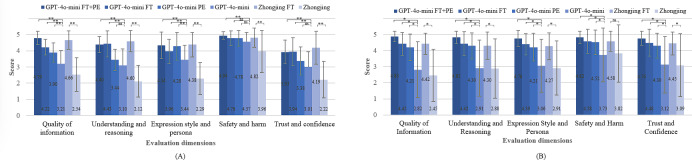
Automatic evaluation (A) and human evaluation (B) of the evaluation agent. Bar heights represent mean scores, and error bars indicate SDs. **P<.01; *P<.05; FT: fine-tuning; ns: not significant; PE: prompt engineering.

Results for the evaluation agent models indicated that, irrespective of whether the model was open source or closed source or whether the evaluation was carried out by humans or through automatic methods, the adapted models consistently surpassed the baseline model in nearly all dimensions (Figure 5; η^2^_p_=0.23‐0.99; Table S7 in Multimedia Appendix 3). In the adaptation process for GPT-4o-mini, both automatic and human evaluations showed that the adaptation strategy of fine-tuning with prompt engineering yielded the highest scores in most dimensions. Specifically, according to both evaluation methods, GPT-4o-mini adapted with fine-tuning and prompt engineering exhibited a substantial improvement in quality of information compared to the fine-tuning–only model (4.79, SD 0.41 vs 4.22, SD 0.48 and 4.88, SD 0.33 vs 4.42, SD 0.71, respectively). In addition, in the automatic evaluation, the GPT-4o-mini fine-tuning with prompt engineering model demonstrated substantially higher scores than GPT-4o-mini with prompt engineering in terms of information quality (4.79, SD 0.41 vs 3.90, SD 0.60), understanding and reasoning (4.40, SD 0.49 vs 3.44, SD 0.66), and trust and confidence (3.93, SD 0.74 vs 3.38, SD 0.86). According to human evaluations, GPT-4o-mini fine-tuning with prompt engineering also outperformed GPT-4o-mini with prompt engineering, scoring substantially higher in information quality (4.88, SD 0.33 vs 4.21, SD 0.82), understanding and reasoning (4.82, SD 0.39 vs 4.30, SD 0.81), and trust and confidence (4.76, SD 0.44 vs 4.30, SD 0.77).

## Discussion

### Principal Findings

In this study, we developed a multi-agent AI system for ACP training and validated its performance through both automatic and expert evaluations. The study yielded several primary implications. First, improvements in model performance were observed for the adapted models when compared to the baseline model. Second, the results underscored the value of synthetic datasets. Third, the effects of different adaptation strategies were inconsistent.

### Comparison to Prior Work

Prior studies have highlighted the challenges associated with using existing LLMs for medical education or training, including limitations in handling ambiguous queries, providing contextually rich responses, and addressing ethical nuances [37]. In comparison, our adapted models demonstrated notable improvements, likely resulting from several key modifications. First, the incorporation of domain-specific datasets [38-40] enabled the models to capture the nuanced linguistic and ethical complexities inherent to the ACP domain. Moreover, advanced adaptation strategies [38,41] improved the models’ ability to understand context, generate empathetic responses, and maintain consistency when addressing sensitive issues. This finding has significant implications for integrating advanced technologies into the realm of ACP.

Synthetic data were used to construct datasets due to limited availability of real-world ACP conversations. Synthetic data offer advantages in scalability, diversity, cost-effectiveness, and privacy protection [42]. They allow for the creation of a broader range of scenarios, including rare or underrepresented cases, thereby improving model generalizability [43,44]. Because data generation can be automated, synthetic data reduce the time, cost, and human annotation burden while avoiding exposure of identifiable patient information [45,46]. However, synthetic data also have limitations. They may reflect biases present in the baseline models and produce more homogenized language compared with natural human dialogue. Furthermore, the emotional intensity, ambiguity, relational dynamics, and cultural nuances of real ACP conversations may not be fully captured in simulated exchanges. To mitigate these risks, data generation was informed by published ACP literature, including clinical case reports and communication examples. During dataset construction, cases reflecting diverse ages, disease types, cultural backgrounds, and psychosocial characteristics were incorporated to increase contextual coverage. An independent LLM was also used as an external evaluator to further validate dataset quality. Nonetheless, the ecological validity and robustness of the system require further evaluation with real-world clinical dialogues and prospective validation before clinical training deployment.

This study found inconsistencies in the effects of different adaptation strategies. Specifically, for the assistant agent, prompt engineering outperformed fine-tuning and even surpassed the combined fine-tuning and prompt engineering approach. Several factors may explain this outcome. First, the knowledge-based dialogue dataset was constructed based on scattered knowledge points; for example, each point typically addressed only a single research topic in ACP. This fragmentation likely diminished the model’s capabilities to capture broader contextual relationships. Second, CoT prompts can substantially improve multistep reasoning and decision-making in generation tasks, giving prompt engineering an edge in tasks requiring explicit chain reasoning. For the remaining 2 agents, the adaptation strategy of fine-tuning with prompt engineering yielded the best performance. However, the adaptation effects of fine-tuning and prompt engineering alone were inconsistent, consistent with prior research. Maharjan et al [47] found that prompt engineering outperformed fine-tuning in medical question answering, whereas Botunac et al [48] reported that fine-tuning models achieved better performance and faster execution. Our experiments revealed that the relative effectiveness of adaptation strategies depended on agent role and task demands rather than showing a single uniformly superior approach.

The application of LLM-based ACP chatbots represents a promising approach to enhancing the accessibility and flexibility of ACP training. LLM-based agents can provide context-specific guidance, simulate diverse clinical scenarios [49], and support dynamic role-playing experiences that may complement traditional training methods. By offering interactive feedback and adaptive responses [39,41], these systems may facilitate experiential learning in a structured and low-risk environment for health care providers. While LLM-based training tools may offer advantages in scalability [50], adoption in resource-limited environments may be constrained by infrastructure gaps, digital literacy variability, and institutional readiness. Deployment requires reliable digital infrastructure, stable internet connectivity, and institutional technical support. Financial considerations may include application programming interface use fees, server hosting, maintenance, and system updates. Additionally, health care providers may require orientation and dedicated training time to effectively integrate the system into existing continuing education or onboarding programs. Further implementation research is needed to evaluate feasibility, cost-effectiveness, and integration pathways within diverse health care systems.

This study was conducted entirely within the Chinese linguistic and health care context, and its findings should be interpreted accordingly. In China, ACP practices are influenced by family-centered decision-making norms, culturally sensitive communication about end-of-life issues, and evolving legal frameworks for advance directives [51,52]. These contextual factors influenced dataset construction, prompt design, and expert evaluation and may differ substantially from ACP models in Western or other health care systems that emphasize individual autonomy and formally established legal structures [53]. Accordingly, the results may not directly generalize to health care systems with different cultural norms, linguistic structures, legal regulations, or ACP training standards. Adaptation to other contexts would require culturally grounded vignette reconstruction, local expert validation, and recalibration of evaluation metrics.

### Limitations

To the best of our knowledge, this study is among the first to develop LLM-powered agents specifically designed to support ACP discussion training for health care providers in the Chinese context. However, the deployment of LLMs in such a value-laden and sensitive domain inevitably raises critical ethical and safety considerations [54-56] that require careful scrutiny. First, the training corpus relied primarily on synthetic data. Synthetic data may not fully capture the emotional nuance and variability of real-world clinical interactions. In addition, some culturally contextualized descriptions (eg, Chinese patients may defer medical decision-making to physicians) were used in synthetic data, which may inadvertently encode cultural stereotypes and limit the generalizability of the findings beyond the Chinese cultural context. Second, the human evaluation panel had a relatively small sample size and a notable demographic imbalance, with 90.9% (10/11) of the experts being female. This may limit the statistical power of the analysis and restrict the generalizability of the human evaluation results. Gender-related differences in communication styles or perceptions of empathy may also have influenced subjective ratings. Third, GPT-4o was used as the automatic evaluator, whereas GPT-4o-mini was one of the adapted models. Given the shared architectural lineage of these 2 models, it could introduce bias into the evaluation results. To mitigate this risk, independent human expert evaluation was incorporated. Fourth, the adaptation process was not fully symmetric across models as prompt engineering was applied only to the closed-source model, meaning that cross-model comparisons should be interpreted with this methodological difference in mind. In addition, adversarial safety testing and formal real-time safeguards such as automated hallucination detection were not implemented in this proof-of-concept phase. Fifth, although the adapted models demonstrated improved performance compared with their baseline versions, their competency cannot yet be directly equated to that of trained health care professionals. The system was assessed using standardized scenarios rather than real-world clinical deployment, and learner outcome measurements were not included.

### Future Directions

This study focused on the development, adaptation, and preliminary evaluation of a multi-agent AI system as a proof-of-concept framework. Future work may incorporate structured red-teaming protocols, automated factuality checks, bias auditing procedures, or human-in-the-loop supervision to comprehensively evaluate safety, ethical alignment, and robustness. Future research will involve prospective validation using real ACP conversations, including small-scale pilot deployment in clinical settings. Comparative studies with traditional training approaches and randomized controlled trials are needed to determine whether the system improves ACP knowledge, confidence, communication competence, and real-world clinical practice outcomes. Such validation will be critical before considering broader educational or clinical implementation. In addition, future research may include cross-cultural validation studies and pilot implementation in diverse health care settings to assess transferability. Such work will be essential before considering international deployment of this multi-agent AI framework.

### Conclusions

In summary, this study developed and technically evaluated a multi-agent LLM-based system designed to simulate ACP conversations for the training of health care providers, ultimately supporting shared decision-making and fostering patient autonomy. The system was adapted using synthetic data generated from prompts grounded in ACP-related literature and policy documents. Both automatic and structured human evaluations indicated that the adapted models outperformed baseline models across multiple dimensions of Chinese ACP dialogue and summarization quality. While these findings demonstrate technical feasibility, the system’s effectiveness in improving health care providers’ real-world ACP competencies requires further validation in educational and clinical settings before broader implementation.

## Supplementary material

10.2196/87288 Multimedia Appendix 1 Prompts.

10.2196/87288 Multimedia Appendix 2 Adaptation configuration.

10.2196/87288 Multimedia Appendix 3 Dialogue example and evaluation.

10.2196/87288 Checklist 1 DEAL checklist.

## Abbreviations

ACP: advance care planning
AI: artificial intelligence
CoT: chain of thought
DEAL: Development, Evaluation, and Assessment of Large Language Models
LLM: large language model
LoRA: low-rank adaptation
QUEST: Quality of Information, Understanding and Reasoning, Expression Style and Persona, Safety and Harm, and Trust and Confidence
