# Supplementary material for: A Large Language Model–Driven System for Advance Care Planning Training Among Health Care Providers in the Chinese Context: Development and Technical Evaluation
Source: J Med Internet Res. 2026 Jul 28;28:e87288. doi: 10.2196/87288 (PMC13411431; doi:10.2196/87288)
Supplement: Multimedia Appendix 2 [file jmir-v28-e87288-s002.docx]

**Appendix 2**

**C1. Zhongjing Model Fine-Tuning Configuration**

| **Parameter** | **Value** |
| --- | --- |
| Base model | Zhongjing-LLaMA-base |
| Training method | LoRA |
| Task type | Causal language modeling |
| LoRA rank (r) | 32 |
| LoRA alpha | 32 |
| LoRA dropout | 0.1 |
| Target modules | q_proj, k_proj, v_proj, o_proj, gate_proj, up_proj, down_proj |
| Training epochs | 10 |
| Learning rate | 7 × 10⁻⁴ |
| Learning rate scheduler | Cosine decay |
| Per-device batch size | 10 |
| Gradient accumulation steps | 4 |
| Effective batch size | 40 |
| Training precision | BF16 mixed precision |

**C2. Supervised Fine-Tuning Configuration for GPT-4o mini**

| **Parameter** | **Value** |
| --- | --- |
| Learning rate | 0.05 |
| Batch size | 2~8 (0.2% × training examples) |
| Training epochs | 10 |
| Temperature | 1.0 |
| Max tokens | 2048 |

The fine-tuned GPT-4o mini model weights cannot be shared due to platform restrictions imposed by the OpenAI API.

**C3. Inference and completion parameters for Zhongjing and ChatGPT-4o**

| **Models** | **Parameter** | **Value** |
| --- | --- | --- |
| Zhongjing | Temperature | 1.0 |
|  | Max tokens | 512 |
| ChatGPT-4o | Temperature | 1.0 |
|  | Max tokens | 2048 |

**C4. Training Monitoring and Convergence**


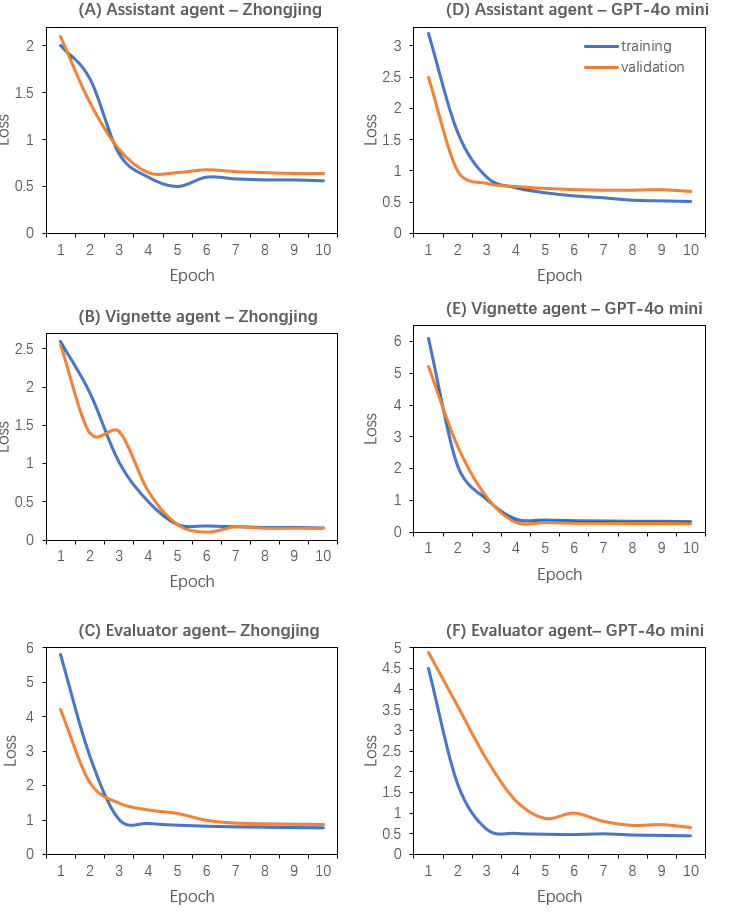


**C5. Computational Resources**

| **Component** | **Specification** |
| --- | --- |
| GPU | 8 × NVIDIA A6000 |
| GPU memory | 48 GB per GPU |
| Training framework | llmtuner, PyTorch  https://github.com/hiyouga/LlamaFactory |

**Approximate training duration**

| **Model** | **Training Time** |
| --- | --- |
| Zhongjing LoRA fine-tuning | ~24 hours |
| GPT-4o mini fine-tuning | managed by API |
