# Supplementary material for: A Large Language Model–Driven System for Advance Care Planning Training Among Health Care Providers in the Chinese Context: Development and Technical Evaluation
Source: J Med Internet Res. 2026 Jul 28;28:e87288. doi: 10.2196/87288 (PMC13411431; doi:10.2196/87288)
Supplement: Multimedia Appendix 3 [file jmir-v28-e87288-s003.docx]

| **Appendix D**  **Table S1** Examples of Knowledge-Based Dialogue | | | |
| --- | --- | --- | --- |
| **Dimension** | **Number** | **Knowledge points (examples)** | **Dialogues (examples)** |
| Overview of ACP | 1117 | Concept of Advance Care Planning: Advance Care Planning (ACP) is a person-centered approach aimed at planning for current and future health and personal care, reflecting an individual’s values, beliefs, and preferences. | **Q:** Hi, I’d like to talk about advance care planning, or ACP. Do you know what that is?  **A:** I’ve heard of it, but I’m not really sure what it means. **Q:** ACP is basically planning ahead for your health and personal care. It’s about making sure your values, beliefs, and preferences are respected, both now and in the future. **A:** Oh, okay. How would it help me? **Q:** It can help you live better, keep some control over your treatment, and make sure your wishes are followed. |
| Common medical decisions in ACP | 171 | Types of Life-Sustaining Treatments: Key discussion points: Whether the patient wants cardiopulmonary resuscitation (CPR) if the heart stops; whether to use mechanical ventilation if they cannot breathe independently; how to manage pain relief; how to address other medical problems and complications. | **Q1:** We should also talk about your preferences for specific treatments. For example, if your heart stops, would you want CPR?  **A1:** What exactly happens with CPR? **Q1:** CPR can save lives, but it can also break ribs or cause other injuries. Even if it works, it might affect your quality of life. We want to know what you’d prefer so we can act accordingly. **A1:** I’d want CPR if it has a good chance of helping, but if the doctor thinks it’s unlikely, then I’d rather not.  **Q2:** If one day you can’t breathe on your own, would you want a breathing machine? **A2:** You mean intubation? **Q2:** Yes, a tube would help you breathe. It can be uncomfortable, and if your illness is severe, it could be needed long-term. **A2:** I’d be okay with it for a short time, but I don’t want to depend on it forever. **Q2:** Got it. We can note that in your advance directive: short-term is okay, long-term dependence is not. |
| Process and Participants in ACP | 2139 | Timing and Frequency of ACP Discussions: The timing and nature of ACP may vary depending on a person’s health status. Whether someone is healthy, has mild-to-moderate chronic conditions, or is a seriously ill patient expected to die within the next 12 months, ACP should be proactive, timely, and integrated into routine care. | **Q:** The right time for ACP depends on your health. Whether you’re healthy or sick, it’s best to start early and make it part of your routine care. **A:** Can I do it now? **Q:** Absolutely. Doing it now helps make sure your future care matches what you want. |
| Social support for ACP | 112 | Roles and Responsibilities of Clinical Healthcare Providers in ACP: Healthcare providers should actively communicate with patients, understand their treatment goals and preferences, and respect the patient’s wishes in treatment decisions. | **Q:** As your healthcare provider, I’ll listen to you, understand your goals, and respect your choices. What do you expect from me? **A:** I hope you’ll be honest and respect my decisions. **Q:** I will. My goal is to make sure you feel supported and understood the whole way. |
| Interpretation of ACP in the context of Chinese culture | 320 | Culture: In China, patients often have limited awareness of their own rights. They may view doctors as authorities and fear making the wrong choice, or, due to family members’ lack of consideration for end-of-life care, allow doctors to act as decision-makers. | **Q:** Many people see doctors as authority figures and worry about making wrong choices. **A:** Does that mean I can decide for myself? **Q:** Yes. ACP helps you know your rights and gives you more control over your care. |
| Life and Death Education | 255 | The Dialectical Relationship Between Life and Death: Advances in medical technology have brought enormous benefits, but for some patients, these benefits may prolong suffering. Especially in the ICU, many terminal patients rely on advanced medical equipment to maintain minimal vital signs, yet ultimately cannot escape death. This not only increases patient suffering but also imposes a significant burden on families. | **Q:** Sometimes, ICU patients rely on machines but still can’t survive. That can cause suffering and stress for families. **A:** Yeah, that worries me too. **Q:** ACP can reduce unnecessary treatments, help patients pass peacefully, and ease the burden on families. |
| Others | 103 | Importance of Home Healthcare Services: Home healthcare (HHC) provides end-of-life care for patients with dementia. By reducing emergency hospitalizations and unnecessary medical transfers, it improves patients’ quality of life. | **Q:** For patients with dementia, home healthcare can improve life quality, reduce emergency visits, and avoid unnecessary hospital transfers. **A:** How does home care work exactly?  **Q:** A team visits regularly to provide medical and nursing support, so patients can get professional care at home. |

| **Table S2** Characteristics of ACP vignettes | | | | | | | |
| --- | --- | --- | --- | --- | --- | --- | --- |
| **Characteristics** |  | **Collected** | **Generated** |  |  | **Collected** | **Generated** |
| **Demographics** | | | | | | | |
| **Age** | ＜20 | 4 | 36 | **Disease** | Hemorrhagic fevers | 0 | 13 |
|  | 20-39 | 2 | 28 |  | Tuberculosis | 0 | 13 |
|  | 40-59 | 15 | 217 |  | HIV/AIDs | 1 | 13 |
|  | 60-79 | 22 | 297 |  | Cancer | 13 | 329 |
|  | 80+ | 4 | 28 |  | Leukemia | 0 | 15 |
|  | Not mentioned | 14 | 4 |  | EMBID | 5 | 13 |
| **Gender** | Male | 26 | 305 |  | Malnutrition | 1 | 13 |
|  | Female | 33 | 305 |  | Dementia | 7 | 15 |
|  | Not mentioned | 2 | 0 |  | Inflammatory disease of CNS | 0 | 13 |
| **Ethnicity** | African | 0 | 24 |  | Degenerative disease of CNS | 1 | 14 |
|  | Asian | 8 | 380 |  | Cerebrovascular diseases | 2 | 13 |
|  | European | 0 | 24 |  | Non-ischemic heart diseases | 0 | 13 |
|  | North American | 1 | 24 |  | Ischemic heart diseases | 5 | 13 |
|  | South American | 1 | 24 |  | Atherosclerosis | 1 | 13 |
|  | Oceania | 1 | 24 |  | Lung diseases | 5 | 15 |
|  | Two or more | 0 | 24 |  | Diseases of liver | 5 | 13 |
|  | Not mentioned | 50 | 86 |  | Musculoskeletal disorders | 1 | 13 |
| **Religion** | Catholicism | 1 | 23 |  | Renal failure | 4 | 15 |
|  | Christianity | 1 | 22 |  | Low birth weight | 0 | 13 |
|  | Buddhism | 0 | 24 |  | Congenital malformations | 1 | 13 |
|  | Taoism | 0 | 22 |  | Injury | 5 | 13 |
|  | Islam | 0 | 23 |  | Healthy | 5 | 12 |
|  | None | 0 | 26 |  | Not mentioned | 4 | 0 |
|  | Other | 0 | 24 |  |  |  |  |
|  | Not mentioned | 59 | 446 |  |  |  |  |
| **Intervention frameworks** | | | | | | | |
| **Transtheoretical Model** |  |  |  |  |  |  |  |
| HCP | 1 | 25 | 135 | D | 1 | 21 | 133 |
|  | 2 | 13 | 128 |  | 2 | 9 | 125 |
|  | 3 | 7 | 123 |  | 3 | 5 | 124 |
|  | 4 | 2 | 118 |  | 4 | 11 | 123 |
|  | 5 | 14 | 106 |  | 5 | 15 | 105 |
| I | 1 | 10 | 125 | A | 1 | 16 | 125 |
|  | 2 | 13 | 126 |  | 2 | 16 | 123 |
|  | 3 | 3 | 126 |  | 3 | 12 | 120 |
|  | 4 | 8 | 123 |  | 4 | 3 | 120 |
|  | 5 | 27 | 110 |  | 5 | 14 | 122 |
| **Behavioural Drivers Model** | Personal characteristics | 21 | 148 | **Behavior Change Techniques** | Goals and planning | 31 | 230 |
|  | Context | 7 | 45 |  | Feedback and monitoring | 12 | 105 |
|  | Cognitive biases | 28 | 175 |  | Social support | 18 | 109 |
|  | Interest | 12 | 153 |  | Shaping knowledge | 20 | 150 |
|  | Attitude | 26 | 203 |  | Natural consequences | 14 | 176 |
|  | Self-efficacy | 21 | 192 |  | Comparison of behaviour | 8 | 95 |
|  | Intent | 2 | 15 |  | Associations | 15 | 151 |
|  | Limited rationality | 7 | 123 |  | Repetition and substitution | 13 | 123 |
|  | Action | 1 | 4 |  | Comparison of outcomes | 18 | 130 |
|  | Behaviour | 0 | 2 |  | Reward and threat | 5 | 98 |
|  | Advocacy | 0 | 0 |  | Regulation | 3 | 61 |
|  | Social influence | 6 | 114 |  | Antecedents | 10 | 69 |
|  | Community dynamic | 1 | 18 |  | Identity | 8 | 84 |
|  | Meta-norms | 7 | 81 |  | Scheduled consequences | 2 | 53 |
|  | Reinforcement | 0 | 0 |  | Self-belief | 14 | 112 |
|  | Communication environment | 2 | 41 |  | Covert learning | 2 | 18 |
|  | Emerging alternatives | 2 | 15 |  |  |  |  |
|  | Governing entities | 2 | 9 |  |  |  |  |
|  | Structural barriers | 3 | 35 |  |  |  |  |

Abbreviations: HCP, health care proxy; D, decision-making flexibility; I, what is most important in life; A, asking doctors questions; EMBID, endocrine, metabolic, blood and immune disorders; CNS, Central Nervous System.

| **Table S3** Examples of Vignette-based Dialogues and Summarization | | |  |
| --- | --- | --- | --- |
| **Clinical case** | **Vignette** | **Healthcare provider-patient dialogues** | **Summarization** |
| Mary had colon cancer two years ago and had surgery to remove the tumor. During a checkup for a cold that wouldn’t go away, doctors found the cancer had come back and spread into her lungs. Mary was told by the doctor that there was no cure.  Mary thought about her father, who died of cancer five years earlier. He traveled back and forth to Vancouver for cancer treatments. One day, her father made the decision to stop cancer treatment and stay at home with his family even though he knew his decision might shorten his life.  At the time, Mary was so angry at her father! She felt like he was giving up. He gathered the family together and spoke quietly.  “I’m tired of all of this,” he said. “I just want to be home with my grandkids. Sleeping in my own bed, eating my traditional food and keeping my own schedule. I don’t want to spend the rest of my life going to doctor appointments. I have a life left to live. I don’t want to waste so much of my time and energy and be too sick to go home. I don’t want to die in the hospital. For what? When it’s my time, it’s my time. This is my decision.”  Mary smiled when she remembered him. He was so happy to be home snuggling with his grandkids on the couch and riding with his son to check the fishing nets. He was enjoying his life and was truly at peace with his choices. He died shortly after the holidays, but Mary was grateful for their time.  It meant so much to her.  She thought about her cancer treatment and wondered how her journey would look. What was important to her? What did she want to do before she got too sick? Where did she want to be? What would she be willing to endure for the sake of more time? And, more importantly, when was enough, enough for her? Mary realized she needed to share her thoughts with her family, and pick a substitute decision maker. | Demographics   - Age: Not specified - Gender: Female - Ethnicity: Not specified - Religious beliefs: Not specified   Health Status   - Diagnosed with colon cancer two years ago and underwent surgical tumor removal. - Recent tests show cancer recurrence with metastasis to the lungs, which is incurable.   Treatment Plan   - Not yet decided. Considering whether to continue treatment or, following her father’s example, stop treatment to improve quality of life.   Needs/Problems   - Facing decisions related to cancer treatment, needing to consider her quality of life and preferred place for end-of-life care. - Wants to discuss with family and choose a healthcare proxy.   ACP Readiness   - Healthcare decision-maker: Considering doing this within the next 6 months (3) - Most important in life: Considering doing this within the next 6 months (3) - Decision flexibility: Considering doing this within the next 6 months (3) - Questions for physician: Considered but not ready (2)   Behavioral Drivers Model Factors   - 12.8 Attitudes and behaviors referenced from networks: Memories of her father’s choice to live at home influence her current decision-making. - 8.8 Decision context/framework: The context and framework for making decisions have a strong impact on the choice of action.   Proposed Behavioral Change Techniques:   - 6.3 Understanding others’ attitudes: Provide perspectives of others on the behavior. - 10.9 Self-reward: Encourage the individual to congratulate or reward herself when progress is made in behavior change. - 15.3 Focus on past successful cases: Suggest recalling or listing previous successful experiences in achieving target behaviors. | **Healthcare Provider:** Hi! I’m glad we can talk today. I’m a healthcare professional here. What would you like to discuss? Any health concerns or questions on your mind? **Patient:** Hello. Yes, I do have some health issues I want to talk about. Two years ago, I had colon cancer and surgery to remove the tumor. But recently, tests showed it’s come back and spread to my lungs. The doctor said it can’t be cured.  **Healthcare Provider:** I’m really sorry to hear that. That must be very hard for you. Have you thought about treatment options yet, or are you still deciding? **Patient:** I haven’t made a specific decision. I’m trying to figure out whether to keep going with treatment or, like my father did, stop treatment and spend my remaining time at home, focusing on quality of life.  **Healthcare Provider:** That sounds like a really important decision. I understand why quality of life is your priority. Have you thought about talking this through with your family or choosing someone to help make healthcare decisions if needed? **Patient:** Yes, I want to talk to my family, maybe in the next six months. I also want to choose a healthcare proxy, but I haven’t decided who yet.  **Healthcare Provider:** That’s an important step. Picking a proxy can help reduce stress and give guidance for you and your family. Have you thought about which family members might be involved or what matters most to you? **Patient:** I’ve been thinking about it. Family support is very important to me, and I want to hear their thoughts on my choices.  **Healthcare Provider:** It sounds like your family has a big role in your thinking. You mentioned your father’s choice—could you tell me more about that? How has it influenced your decisions? **Patient:** My father chose to spend his final days at home rather than continue treatment. I respect that a lot, and it’s making me think carefully about whether I want to continue treatment or follow a similar path.  **Healthcare Provider:** That’s completely understandable. Being at home can provide more comfort and dignity, especially at the end of life. Everyone’s situation is different. Besides your family, have you talked to your doctor about treatment options, or thought about what questions to ask? **Patient:** I’ve thought about it, but I’m not ready yet. I feel hesitant to ask more questions.  **Healthcare Provider:** That’s okay. It can feel overwhelming, but talking with your doctor helps you understand your options. Some people also find it useful to hear how others made similar decisions. I can share a few patient stories if you’d like. **Patient:** Yes, that would help. I think learning from others could guide me.  **Healthcare Provider:** Here are a couple of examples:   - A 65-year-old man with late-stage pancreatic cancer decided to stop treatment after chemotherapy because it was too hard on him physically and emotionally. He went home for palliative care, spent time with his family, and chose his wife as his healthcare proxy. This gave him and his family peace of mind. - A 72-year-old woman with late-stage lung cancer chose to continue immunotherapy to extend her life while maintaining some quality of life. She discussed her wishes clearly with her family and named her son as proxy if her condition worsened. Planning ahead gave her and her family a sense of control.   **Patient:** I see. Both of them made choices that suited them, and family support was important.  **Healthcare Provider:** Exactly. The main point is to make decisions that fit your values and communicate them clearly to your family and healthcare team. Also, think about past experiences where you made tough decisions. For example, your surgery was a brave choice. Remembering that can give you confidence now. **Patient:** That’s true. I was determined with my surgery.  **Healthcare Provider:** Right. Your resilience then shows you have the strength to make decisions now. Whether you continue treatment or focus on comfort at home, you can make choices aligned with your values.  **Healthcare Provider:** From our talk, it seems like you’ve started thinking seriously about your future plans, like discussing with family and choosing a healthcare proxy. Which of these would you like to focus on next? **Patient:** I think talking with my family comes first, then I can think about discussing more with my doctor.  **Healthcare Provider:** That’s a good approach. Take it step by step. I can provide some tips and resources on how to talk with your family about your wishes. The important thing is to communicate openly, and you’ll find a path that works best for you. **Patient:** Thank you. That’s really helpful. | **Overall Feedback:** After reviewing the dialogue, it is clear that you demonstrated a strong understanding of ACP and effectively employed communication skills to facilitate the patient’s comprehension and self-expression when discussing major medical decisions. You sensitively identified the patient’s hesitation regarding future treatment choices and the influence of family factors, while also guiding the patient to reflect on past experiences and personal values, helping them gradually clarify their decision-making direction.  **Key Strengths:**   1. **Conveying Core ACP Knowledge:** You successfully highlighted essential elements of ACP, including attention to quality of life, the importance of selecting a healthcare proxy, and the influence of family on patient decisions. This helped the patient understand ACP and its relevance to their own situation. 2. **Assessing Patient ACP Readiness:** You effectively recognized and responded to the patient’s considerations about family support and medical decisions, noting that the patient had not yet discussed these issues in depth with their physician. You also guided the patient to reflect on choosing a healthcare proxy, providing meaningful support for future decision-making. 3. **Flexible Communication Skills:** You skillfully used open-ended questions and empathetic responses to help the patient explore their values and expectations for the future. Additionally, by sharing case examples, you helped the patient understand how others navigated similar situations, supporting a more comprehensive consideration of their own choices.   **Key Areas for Improvement:**   1. **Application of Behavioral Change Techniques:** While you used strategies such as case-based guidance, there is room to expand the range of behavioral change techniques. Incorporating planning and goal-setting strategies could provide the patient with clearer direction and motivation when preparing to discuss ACP with family or physicians. 2. **Cultural Sensitivity:** Cultural aspects of ACP were not deeply addressed. Integrating the patient’s cultural background and beliefs about life and death could provide more culturally congruent support during future planning. 3. **Guiding the Patient to Set Clear Short-Term Goals:** Although you encouraged family discussions, assisting the patient in defining specific, time-bound action steps would enhance their confidence and readiness for ACP.   **Future Development:** To further strengthen your ACP knowledge and skills:   1. **Expand Behavioral Change Techniques:** In future dialogues, consider incorporating additional strategies such as “association cues” and “monitoring/feedback” to more systematically support patient understanding and decision-making in ACP discussions. 2. **Enhance Cultural Responsiveness:** Explore ways to integrate patients’ cultural values and beliefs into ACP conversations to ensure guidance is aligned with their worldview. 3. **Support Goal-Setting:** Encourage patients to define specific short-term actions with clear timelines, helping them translate discussions into practical steps toward ACP readiness. |

| **Table S4** Strengths and limitations in the summarization dataset | | | |
| --- | --- | --- | --- |
| **Strengths** | | **Limitations** | |
| **Dimension** | **N** | **Dimension** | **N** |
| Communication skills | 581 | ACP-related knowledge | 520 |
| ACP-related knowledge | 550 | Application of behavior change techniques | 410 |
| Application of behavior change techniques | 506 | Enhance the interpretation of ACP in the context of Chinese culture | 393 |
| Readiness assessment | 441 | Readiness assessment | 192 |
| Empathy | 118 | Guide patient thinking | 97 |
| Emotional support | 94 | Promote decision-making | 92 |
| Guide patient thinking | 79 | Strengthen family and social support | 88 |
| Provide information and resources | 78 | Communication skills | 59 |
| Values discussion | 58 | Values discussion | 57 |
| Promote patient/family participation | 52 | Emotional support | 39 |
| Supportive feedback | 33 | Continuous feedback | 39 |
| Promote decision-making | 31 | Provide information and resources | 38 |
| Focus on patient needs | 24 | Life and death education | 24 |
| Effective listening | 19 | Enhance patient confidence | 23 |
| Cultural sensitivity | 14 | Assess influencing factors | 10 |
| **Avg Points Per Summarization** | 3.99 | **Avg Points Per Summarization** | 3.10 |

| **Table S5** Automatic and human evaluation of assistant agents | | | | | | |  |
| --- | --- | --- | --- | --- | --- | --- | --- |
| **Evaluator** | **Model** | **Quality of Information** | **Understanding and Reasoning** | **Expression Style and Persona** | **Safety and Harm** | **Trust and Confidence** | |
| **Automatic** | GPT-4o mini FT+PE ^1^ | 4.35±0.51 | 4.12±0.66 | 4.04±0.75 | 4.90±0.30 | 4.26±0.51 | |
| (n=436) | GPT-4o mini FT ^2^ | 4.19±0.80 | 4.10±0.88 | 3.79±0.75 | 4.87±0.37 | 4.09±0.86 | |
|  | GPT-4o mini PE ^3^ | 4.65±0.71 | 4.58±0.81 | 4.51±0.77 | 4.97±0.18 | 4.64±0.71 | |
|  | GPT-4o mini ^4^ | 3.16±1.00 | 2.95±1.06 | 3.17±0.83 | 4.56±0.81 | 3.01±1.09 | |
|  | Zhongjing FT ^5^ | 3.69±0.78 | 3.53±0.90 | 3.57±0.70 | 4.80±0.46 | 3.57±0.86 | |
|  | Zhongjing ^6^ | 3.01±0.76 | 2.85±0.77 | 3.02±0.74 | 4.60±0.61 | 2.94±0.79 | |
|  | F | 319.43 | 277.10 | 230.00 | 57.09 | 304.95 | |
|  | df_1_ | 4.36 | 4.38 | 4.38 | 3.09 | 4.27 | |
|  | df_2_ | 1899.42 | 1907.58 | 1910.64 | 1346.23 | 1861.89 | |
|  | P | ＜0.01 | ＜0.01 | ＜0.01 | ＜0.01 | ＜0.01 | |
|  | η²ₚ | 0.42 | 0.39 | 0.35 | 0.12 | 0.41 | |
|  | Intra-group comparison |  |  |  |  |  | |
|  | Baseline and adapted models | 3, 1, 2＞4,  5＞6 | 3, 1, 2＞4,  5＞6 | 3, 1, 2＞4,  5＞6 | 3, 1, 2＞4,  5＞6 | 3, 1, 2＞4,  5＞6 | |
|  | GPT-4o mini models at different adaptation process | 3＞1＞2 | 3＞1=2 | 3＞1＞2 | 3＞1=2 | 3＞1＞2 | |
|  | Open-source and closed-source models before and after adaptation | 4=6, 2＞5 | 4=6, 2＞5 | 4=6, 2＞5 | 4=6, 2=5 | 4=6, 2＞5 | |
| **Human** | GPT-4o mini FT+PE ^1^ | 4.35±0.84 | 4.36±0.89 | 4.35±0.80 | 4.62±0.73 | 4.22±0.98 | |
| (n=5) | GPT-4o mini FT ^2^ | 4.18±1.02 | 4.05±1.06 | 3.98±1.05 | 4.31±1.03 | 4.04±1.22 | |
|  | GPT-4o mini PE ^3^ | 4.69±0.79 | 4.64±0.80 | 4.58±0.74 | 4.73±0.78 | 4.69±0.90 | |
|  | GPT-4o mini ^4^ | 2.55±1.71 | 2.71±1.77 | 2.84±1.72 | 3.16±1.84 | 2.51±1.68 | |
|  | Zhongjing FT ^5^ | 3.45±1.17 | 3.56±1.29 | 3.55±1.17 | 4.00±1.25 | 3.49±1.32 | |
|  | Zhongjing ^6^ | 1.55±1.14 | 1.85±1.50 | 1.95±1.53 | 2.53±1.81 | 1.53±1.14 | |
|  | F | 22.15 | 24.20 | 26.50 | 27.63 | 34.00 | |
|  | df_1_ | 2.08 | 1.70 | 1.88 | 1.60 | 1.68 | |
|  | df_2_ | 8.30 | 6.78 | 7.50 | 6.38 | 6.71 | |
|  | P | ＜0.01 | 0.01 | ＜0.01 | 0.01 | ＜0.01 | |
|  | η²ₚ | 0.85 | 0.86 | 0.87 | 0.87 | 0.90 | |
|  | Intra-group comparison |  |  |  |  |  | |
|  | Baseline and adapted models | 3, 1, 2＞4 , 5＞6 | 3, 1, 2＞4 , 5＞6 | 3, 1＞4 , 5＞6 | 3, 1, 2＞4 , 5＞6 | 3, 1, 2＞4 , 5＞6 | |
|  | GPT-4o mini models at different adaptation process | 3=1=2 | 3=1＞2 | 3=1＞2 | 3=1＞2 | 3＞1=2 | |
|  | Open-source and closed-source models before and after adaptation | 4=6, 2=5 | 4=6, 2=5 | 4=6, 2=5 | 4=6, 2=5 | 4＞6, 2=5 | |

| **Table S6** Automatic and human evaluation of vignette agents | | | | | | |  |
| --- | --- | --- | --- | --- | --- | --- | --- |
| **Evaluator** | **Model** | **Quality of Information** | **Understanding and Reasoning** | **Expression Style and Persona** | **Safety and Harm** | **Trust and Confidence** | |
| **Automatic** | GPT-4o mini FT+PE ^1^ | 4.50±0.50 | 4.60±0.49 | 4.47±0.50 | 4.90±0.31 | 4.49±0.50 | |
| (n=67) | GPT-4o mini FT ^2^ | 3.84±0.59 | 3.72±0.62 | 3.65±0.77 | 4.63±0.54 | 3.76±0.71 | |
|  | GPT-4o mini PE ^3^ | 4.46±0.78 | 4.46±0.72 | 4.51±0.68 | 4.75±0.50 | 4.53±0.70 | |
|  | GPT-4o mini ^4^ | 3.26±0.73 | 2.99±0.87 | 2.99±0.72 | 4.57±0.56 | 2.97±0.85 | |
|  | Zhongjing FT ^5^ | 3.54±0.70 | 3.29±0.71 | 3.40±0.96 | 4.49±0.59 | 3.25±0.66 | |
|  | Zhongjing ^6^ | 3.06±0.71 | 2.91±0.82 | 2.53±0.59 | 4.26±0.68 | 2.79±0.76 | |
|  | F | 54.88 | 73.24 | 87.20 | 14.28 | 80.21 | |
|  | df_1_ | 4.35 | 3.81 | 4.08 | 3.83 | 4.30 | |
|  | df_2_ | 291.65 | 255.46 | 273.07 | 256.60 | 287.82 | |
|  | P | ＜0.01 | ＜0.01 | ＜0.01 | ＜0.01 | ＜0.01 | |
|  | η²ₚ | 0.45 | 0.52 | 0.57 | 0.18 | 0.55 | |
|  | Intra-group comparison |  |  |  |  |  | |
|  | Baseline and adapted models | 1, 3, 2＞4, 5＞6 | 1, 3, 2＞4, 5=6 | 3, 1, 2＞4, 5＞6 | 1＞4, 5=6 | 3, 1, 2＞4, 5＞6 | |
|  | GPT-4o mini models at different adaptation process | 1=3＞2 | 1=3＞2 | 3=1＞2 | 1=3, 1＞2 | 3=1＞2 | |
|  | Open-source and closed-source models before and after adaptation | 4=6, 2=5 | 4=6, 2＞5 | 4＞6, 2=5 | 4＞6, 2=5 | 4=6, 2＞5 | |
| **Human** | GPT-4o mini FT+PE ^1^ | 4.60±0.73 | 4.64±0.71 | 4.39±0.84 | 4.65±0.64 | 4.45±0.85 | |
| (n=10) | GPT-4o mini FT ^2^ | 4.19±0.98 | 4.23±0.97 | 4.14±1.01 | 4.40±1.02 | 4.14±1.10 | |
|  | GPT-4o mini PE ^3^ | 4.38±0.83 | 4.35±0.86 | 4.10±0.94 | 4.42±0.82 | 4.27±0.85 | |
|  | GPT-4o mini ^4^ | 3.10±1.55 | 3.21±1.60 | 3.19±1.49 | 3.95±1.50 | 3.22±1.61 | |
|  | Zhongjing FT ^5^ | 3.74±1.46 | 3.79±1.42 | 3.74±1.39 | 4.11±1.32 | 3.75±1.49 | |
|  | Zhongjing ^6^ | 2.90±1.66 | 3.06±1.64 | 2.99±1.57 | 3.71±1.57 | 2.99±1.70 | |
|  | F | 23.98 | 29.73 | 23.98 | 14.76 | 19.53 | |
|  | df_1_ | 2.43 | 2.22 | 2.18 | 2.03 | 2.32 | |
|  | df_2_ | 21.88 | 20.01 | 19.64 | 18.26 | 20.91 | |
|  | P | ＜0.01 | ＜0.01 | ＜0.01 | ＜0.01 | ＜0.01 | |
|  | η²ₚ | 0.73 | 0.77 | 0.73 | 0.62 | 0.69 | |
|  | Intra-group comparison |  |  |  |  |  | |
|  | Baseline and adapted models | 1, 3, 2＞4, 5＞6 | 1, 3, 2＞4, 5＞6 | 1, 2, 3＞4, 5＞6 | 1, 3, 2＞4, 5=6 | 1, 3, 2＞4, 5＞6 | |
|  | GPT-4o mini models at different adaptation process | 1＞3=2 | 1＞3=2 | 1＞2=3 | 1＞3=2 | 1＞3=2 | |
|  | Open-source and closed-source models before and after adaptation | 4=6, 2=5 | 4=6, 2=5 | 4=6, 2=5 | 4=6, 2=5 | 4=6, 2=5 | |

| **Table S7** Automatic and human evaluation of evaluator agents | | | | | | |  |
| --- | --- | --- | --- | --- | --- | --- | --- |
| **Evaluator** | **Model** | **Quality of Information** | **Understanding and Reasoning** | **Expression Style and Persona** | **Safety and Harm** | **Trust and Confidence** | |
| **Automatic** | GPT-4o mini FT+PE ^1^ | 4.79±0.41 | 4.40±0.49 | 4.34±0.82 | 4.94±0.24 | 3.93±0.74 | |
| (n=67) | GPT-4o mini FT ^2^ | 4.22±0.48 | 4.43±0.80 | 3.96±0.74 | 4.78±0.45 | 3.94±0.86 | |
|  | GPT-4o mini PE ^3^ | 3.90±0.60 | 3.44±0.66 | 4.28±0.84 | 4.78±0.48 | 3.38±0.86 | |
|  | GPT-4o mini ^4^ | 3.21±0.80 | 3.10±1.02 | 3.44±0.90 | 4.57±0.56 | 3.01±0.94 | |
|  | Zhongjing FT ^5^ | 4.66±0.56 | 4.60±0.65 | 4.38±0.75 | 4.82±0.60 | 4.19±1.01 | |
|  | Zhongjing ^6^ | 2.54±1.04 | 2.12±0.99 | 2.29±0.99 | 3.96±1.30 | 2.22±1.13 | |
|  | F | 118.25 | 102.63 | 66.77 | 19.78 | 46.38 | |
|  | df_1_ | 3.71 | 3.97 | 5 | 2.06 | 3.36 | |
|  | df_2_ | 248.49 | 266.08 | 335 | 138.13 | 224.84 | |
|  | P | ＜0.01 | ＜0.01 | ＜0.01 | ＜0.01 | ＜0.01 | |
|  | η²ₚ | 0.64 | 0.61 | 0.50 | 0.23 | 0.41 | |
|  | Intra-group comparison |  |  |  |  |  | |
|  | Baseline and adapted models | 1, 2, 3＞4, 5＞6 | 2, 1＞4, 5＞6 | 1, 3, 2＞4, 5＞6 | 1＞4, 5＞6 | 2, 1＞4, 5＞6 | |
|  | GPT-4o mini models at different adaptation process | 1＞2＞3 | 2=1＞3 | 1=3=2 | 1=2=3 | 2=1＞3 | |
|  | Open-source and closed-source models before and after adaptation | 4＞6, 1=5 | 4＞6, 2=5 | 4＞6, 1=5 | 4＞6, 1=5 | 4＞6, 2=5 | |
| **Human** | GPT-4o mini FT+PE ^1^ | 4.88±0.33 | 4.82±0.39 | 4.76±0.50 | 4.82±0.39 | 4.76±0.44 | |
| (n=3) | GPT-4o mini FT ^2^ | 4.42±0.71 | 4.42±0.75 | 4.39±0.66 | 4.58±0.75 | 4.48±0.83 | |
|  | GPT-4o mini PE ^3^ | 4.21±0.82 | 4.30±0.81 | 4.21±0.86 | 4.52±0.76 | 4.30±0.77 | |
|  | GPT-4o mini ^4^ | 2.82±1.74 | 2.91±1.74 | 3.06±1.64 | 3.73±1.66 | 3.12±1.75 | |
|  | Zhongjing FT ^5^ | 4.42±0.66 | 4.30±0.85 | 4.27±0.84 | 4.58±0.61 | 4.45±0.71 | |
|  | Zhongjing ^6^ | 2.45±1.62 | 2.88±1.85 | 2.91±1.68 | 3.82±1.78 | 3.09±1.96 | |
|  | F | 166.22 | 65.50 | 56.47 | 39.84 | 45.68 | |
|  | df_1_ | 1.92 | 1.98 | 1.75 | 1.91 | 1.29 | |
|  | df_2_ | 3.84 | 3.96 | 3.50 | 3.83 | 2.58 | |
|  | P | ＜0.01 | 0.01 | 0.02 | 0.03 | 0.01 | |
|  | η²ₚ | 0.99 | 0.97 | 0.97 | 0.95 | 0.96 | |
|  | Intra-group comparison |  |  |  |  |  | |
|  | Baseline and adapted models | 1, 2, 3＞4, 5＞6 | 1, 2, 3＞4, 5＞6 | 1, 2, 3＞4, 5＞6 | 1, 2, 3＞4, 5=6 | 1, 2, 3＞4, 5＞6 | |
|  | GPT-4o mini models at different adaptation process | 1＞2, 2=3 | 1＞2, 2=3 | 1=2=3 | 1=2=3 | 1＞2, 2=3 | |
|  | Open-source and closed-source models before and after adaptation | 4=6, 2=5 | 4=6, 2=5 | 4=6, 2=5 | 4=6, 2=5 | 4=6, 2=5 | |
