# Supplementary material for: A Large Language Model–Driven System for Advance Care Planning Training Among Health Care Providers in the Chinese Context: Development and Technical Evaluation
Source: J Med Internet Res. 2026 Jul 28;28:e87288. doi: 10.2196/87288 (PMC13411431; doi:10.2196/87288)
Supplement: Checklist 1 [file jmir-v28-e87288-s004.docx]

**Development, Evaluation, and Assessment of Large Language Models (DEAL) Checklist**

| Section / Topic | No. | Item | Description | Page Number | No | N/A |
| --- | --- | --- | --- | --- | --- | --- |
| Model Information & Study Setup | 1.1 | LLM Model Information | Provide name(s), version(s), and dates of use. Include all models tested. | 10-11 |  |  |
|  | 1.2 | Model Selection Rationale | Explain the rationale behind model selection. | 10-11 |  |  |
|  | 1.3 | Completion Parameters | List parameters such as temperature, max tokens penalties, and logit bias. | Appendix C |  |  |
|  | 1.4 | Custom instructions & Preprocessing | Describe any custom instructions (e.g., "You are...") and preprocessing steps. | Appendix B |  |  |
|  | 1.5 | Most Recent Training Date | Provide the most recent training date of the LLM model used. | 11 |  |  |
|  | 1.6 | Annotation Process | Outline any annotation processes including team roles, guidelines, and quality control measures. | 10 |  |  |
|  | 1.7 | Compute Resources | Specify compute resources (e.g.GPU/CPU hours, cost) used in model training or fine-tuning | Appendix C |  |  |
|  | 1.8 | Ethical Considerations | Indicate Institutional Review Board (IRB) or ethics committee approvals, and patient  consent/waivers, as applicable. | 16 |  |  |
|  | 1.9 | Funding and Disclosures | Disclose funding sources and any potential conflicts of interest (COl). | 29 |  |  |
| Data Handling and Quality Control | 2.1 | Data Sources | Provide detailed information on datasets, including synthetic data private data, or any proprietary data, if applicable. | 7 |  |  |
|  | 2.2 | Data Preparation | Describe cleaning, transformations, and partitioning of the data. | 7-10 |  |  |
|  | 2.3 | Data Quality Checks | Describe procedures for assessing and ensuring data quality. | 7-10 |  |  |
|  | 2.4 | Missing/Imbalanced Data | Document techniques for handling missing data, imbalances, or other data anomalies. | 7-10 |  |  |
|  | 2.5 | Data  Leakage/Contamination | Outline strategies for identifying and mitigating data leakage, especially for closed models where verification is challenging. | 10 |  |  |
| Training, Fine-  Tuning &Testing* | 3.1 | Fine-Tuning Approach | Specify whether parameter-efficient fine-tuning (e.g., LoRA) or full-parameter fine-tuning was applied. | 10-11 |  |  |
|  | 3.2 | Hyperparameters &  Tuning | List the hyperparameters and their values (e.g.,learning rate, r, target_modules). | Appendix C |  |  |
|  | 3.3 | Training Method &Tools | Describe the fine-tuning method (e.g., RLHF, SFT) and the tools (e.g.. PyTorch, TensorFlow). | Appendix C |  |  |
|  | 3.4 | Evaluation Data | Describe the evaluation dataset and testing methodology (e.g..train/test split). | 10 |  |  |
|  | 3.5 | Evaluation Metrics | List metrics used to assess performance (e.g.. accuracy, F1.score, AUROC, BLEU). | 13-14 |  |  |
| Embedding &  Retrieval* | 4.1 | Embedding Creation | Describe embedding creation (e.g..  chunk size, overlap). | N/A |  |  |
|  | 4.2 | Retrieval Techniques | Explain tools and methods for retrieval (e.g., FASS, rerank, compression). | N/A |  |  |
|  | 4.3 | Number of Queries per Prompt | Explain the approach for handling multiple queries in RAG setups to manage model stochasticity. | N/A |  |  |
|  | 4.4 | Data Retrieval Checks | Describe processes for verifying relevance of retrieved data and managing stochastic behavior in retrievals. | N/A |  |  |
|  | 4.5 | Evaluation of Consistency | Indicate metrics or approaches used to evaluate consistency of responses in RAG setups. | N/A |  |  |
| Prompting &Output Evaluation | 5.1 | Prompt Engineering Techniques | Provide comprehensive list of prompts and techniques used (e.g., Chain of Thought). | 12-13 |  |  |
|  | 5.2 | Output Evaluation | Describe how model outputs were evaluated, and list specific metrics for assessment. | 13-14 |  |  |
| Multi-Agent Systems* | 6.1 | Multi-Agent Setup &Coordination | Describe coordination mechanisms and agents used (e.g., Auto Gen, custom coordination). | 6-7 |  |  |
|  | 6.2 | Agent Parameters &Testing | List parameters and distinct strategies used for each agent, if applicable. | Appendix C |  |  |
|  | 6.3 | Agent Coordination and Independence | Define how agents coordinate and handle independence or role specialization. | Appendix C |  |  |
|  | 6.4 | Agent Evaluation and Relevance Checks | Add prompts to assess output quality and relevance for each agent. | 13-14 |  |  |
|  | 6.5 | Agents Stochasticity Management | Detail how stochastic behaviors are monitored and managed across agents for consistency. | N/A |  |  |
| Fairness Evaluation | 7.1 | Bias ldentification Strategy | Describe methods used to detect bias in model outputs, including metrics (e.g., demographic parity) and target groups assessed for fairness. | 13-14 |  |  |
|  | 7.2 | Synthetic Data Bias Evaluation | Explain steps to identify and mitigate biases in studies using synthetic data, including validation for representational fairness. | 16-18 |  |  |
|  | 7.3 | Mitigation Techniques | Specify techniques applied to reduce bias, such as debiasing algorithms or diverse dataset inclusion. | 7-10 |  |  |
|  | 7.4 | Fairness Metrics | List fairness metrics used to assess and report model fairness(e.g., disparate impact ratio, equal opportunity difference). | N/A |  |  |
| Model Stochasticity Management | 8.1 | Multiple Query Consistency Checks | Document approach for managing stochastic outputs, including the number of iterations and analysis of response variations. | N/A |  |  |
|  | 8.2 | Variation Recording and Reporting | Outline methods used to log and report output differences across queries, noting significant deviations. | Appendix D |  |  |
|  | 8.3 | Stochastic Control Techniques | Describe techniques to reduce output variability (e.g., averaging outputs over multiple runs). | 13-14 |  |  |
|  | 8.4 | Consistency Metrics | List metrics or qualitative methods used to assess output consistency,  such as standard deviation or stability checks. | Appendix D |  |  |
